# Effect of Static Posture on Online Performance of P300-Based BCIs for TV Control

**DOI:** 10.3390/s21072278

**Published:** 2021-03-24

**Authors:** Dojin Heo, Minju Kim, Jongsu Kim, Yun-Joo Choi, Sung-Phil Kim

**Affiliations:** Department of Biomedical Engineering, Ulsan National Institute of Science and Technology, Ulsan 44919, Korea; soii0203@unist.ac.kr (D.H.); mjkim28@unist.ac.kr (M.K.); kjstn737@unist.ac.kr (J.K.); joo618@unist.ac.kr (Y.-J.C.)

**Keywords:** brain–computer interface, P300, N200, static posture, comfort

## Abstract

To implement a practical brain–computer interface (BCI) for daily use, continuing changes in postures while performing daily tasks must be considered in the design of BCIs. To examine whether the performance of a BCI could depend on postures, we compared the online performance of P300-based BCIs built to select TV channels when subjects took sitting, recline, supine, and right lateral recumbent postures during BCI use. Subjects self-reported the degrees of interference, comfort, and familiarity after BCI control in each posture. We found no significant difference in the BCI performance as well as the amplitude and latency of P300 and N200 among the four postures. However, when we compared BCI accuracy outcomes normalized within individuals between two cases where subjects reported relatively more positively or more negatively about using the BCI in a particular posture, we found higher BCI accuracy in those postures for which individual subjects reported more positively. As a result, although the change of postures did not affect the overall performance of P300-based BCIs, the BCI performance varied depending on the degree of postural comfort felt by individual subjects. Our results suggest considering the postural comfort felt by individual BCI users when using a P300-based BCI at home.

## 1. Introduction

A brain–computer interface (BCI) enables people to communicate with the external world by conveying the translation of brain activity directly without passing through a normal output pathway involving the utilization of muscles for delivering a user’s commands or message [1]. For non-invasive BCIs based on scalp electroencephalography (EEG), commonly used types of features include a steady-state visual evoked potential (SSVEP), an event-related potential (ERP), and sensorimotor rhythms (SMRs). Among them, P300 is one of the ERP components appearing in a time range from 275 to 600 ms after stimulus onset, especially over central and parietal areas. P300 is induced in association with task-relevant and rare stimuli presented between a series of task-irrelevant and frequent stimuli [2]. A P300-based BCI does not require the learning of self-regulation of brain responses for those who are not familiar with the system [3] and has been one of the most popular forms of non-invasive BCIs [4,5,6] since Farwell and Donchi developed the P300 speller [7].

As the main purpose of BCIs has been to provide means of communication and controlling devices for people with motor disabilities, the application of BCIs in a real-life environment out of a laboratory needs to be taken into consideration. The use of BCIs in daily living environments is substantially different from conducting BCI experiments in a laboratory in several aspects [8]. Likewise, one of the differences arises from a posture taken when using a BCI. People are likely to take various postures (e.g., lying and sitting) when they use a BCI in daily living environments, whereas participants in BCI experiments are typically comfortably seated on an armchair in an isolated environment.

Previous studies reported that a change in postures while performing a single task caused physiological changes in the brain, including cerebrospinal fluid (CSF) [9], the hemodynamic state [10,11,12,13], and shifts in the brain tissues [14,15]. Rice and colleagues disclosed that changing from prone to supine postures made a difference in the thickness of CSF between the scalp and brain due to gravity. They reported differences in various EEG signals such as slow flash-visual evoked potential (F-VEP), oddball ERPs (e.g., P300), steady-state visual evoked potential (SSVEP), as well as spectral power in alpha and gamma bands, in the sitting, supine, and prone positions [9]. Fardo et al. demonstrated a difference in an anterior late positive potential (LPP), which was known to be related to pain perception, between the lying and sitting positions when the same pain stimulation was given and suggested that the lying position could alleviate frontoparietal pain processing [16]. Additionally, Spironelli et al. showed that the power of delta and alpha EEG oscillations, which indexed cortical inhibition in the awakening state without engagement in specific cognitive tasks, differed between the lying and sitting positions, and noted that this difference was related to changes in blood pressure and arterial baroreceptor firing rates [17]. These changes in EEG, especially in ERPs, present possibilities of differences in the ERP characteristics for P300-based BCIs depending on postures, which in turn results in a potential variation in the performance of P300-based BCIs for real-life applications. 

Despite these possibilities, to the best of our knowledge, few studies considered posture as a potential factor when designing a BCI. Several studies demonstrated the feasibility of using BCIs in a real-life environment for patients suffering from amyotrophic lateral sclerosis (ALS) [18,19], in which the users controlled devices through BCIs while lying on the bed or sitting on the wheelchair. However, these studies did not examine whether the change in body positions could affect the BCI performance.

Up to date, only a few studies have investigated the effect of change in body positions on BCI performance. For example, a study on the application of the P300-based BCI in a real-life investigated whether the BCI could be used while moving. It revealed that the control of a mobile P300-based BCI during walking reduced visual attention [20]. Lotte et al. compared the performance of the P300-based BCI among the sitting, standing, and walking conditions, and reported that not every subject performed better in sitting than in standing condition [21]. Not only in the P300-based BCI but also in the SSVEP-based BCI, a study related to the effect of sitting and standing conditions also reported that performance in standing was slightly higher than in sitting [22]. These studies largely focused on the comparison of BCIs performance in sitting condition with other standing and walking conditions. However, the effect of different static postures on the performance of BCIs remains unknown.

Thus, in this study, we aimed to examine the effect of different static postures on the utilization of a P300-based BCI considering using the BCI system in real-life environments. We aimed at clarifying whether changes in postures cause a difference in the ERP characteristics and consequently affect BCI performance. To address this question, we compared online BCI performance when healthy subjects controlled a TV using a P300-based BCI in four different postures (sitting, recline, supine, and right lateral recumbent postures). Additionally, we analyzed the ERP components induced when using the BCI from different postures and the subjective reports obtained from the subjects regarding comforts in each posture. 

## 2. Methods

### 2.1. Subjects

Fourteen healthy subjects (11 male, ages 18–25 years old with mean 22.8 ± 2.04) participated in the experiment. Considering the length of a foldaway bed used in the experiment, subjects whose heights were between 162 and 173 cm participated in the study (167.8 ± 3.13 cm). All subjects have never had any neurological or mental illness and they had normal or were corrected to normal vision. One subject responded to have a joint-related disease (i.e., a disc), but not so severe as to be disturbing to do daily tasks. All subjects gave informed consent for this study, which was approved by the Ulsan National Institute of Science and Technology, Institutional Review Board (UNIST-IRB-18-08-A).

### 2.2. Posture

**Figure 1:**
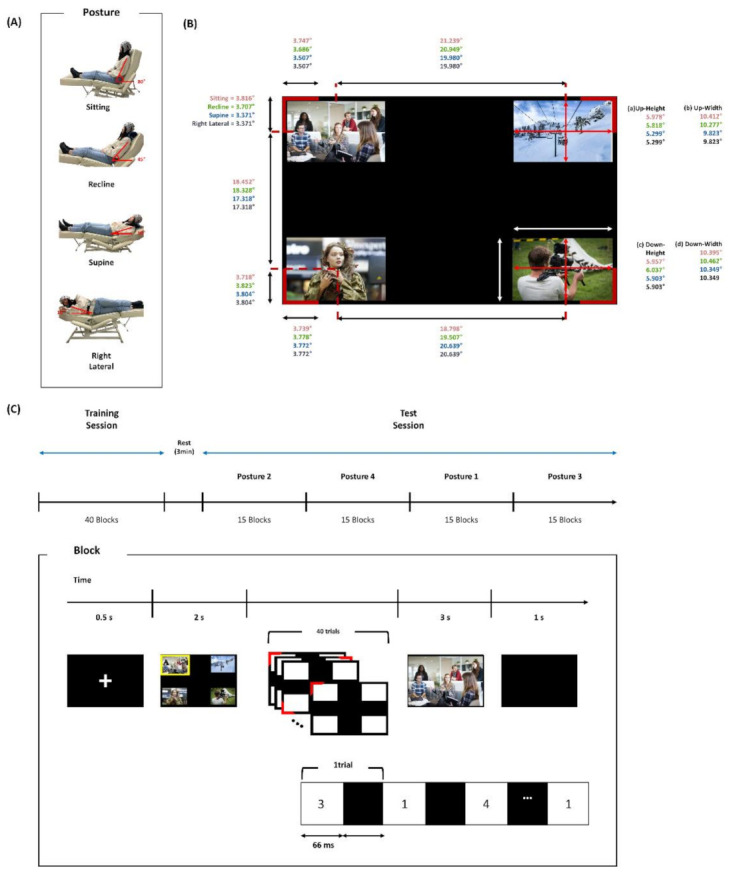
(**A**) Types of postures used in this experiment: sitting, recline, supine, and right lateral posture. The red angles denote the size of the angle between a vertical axis on a sagittal plane and a flat floor for sitting, recline, supine, and right lateral postures. (**B**) Visual stimuli and visual angles for each stimulus in each posture. The red text indicates the visual angle in the sitting posture, the green in the reclining posture, the blue in the supine, and the black is in the right lateral recumbent posture. The visual angles for the height and width of the presented video clip at the upper side (a,b) and lower side (c,d) are described next to the visual stimulus. The represented image of the video clips was replaced by that of copyright-free videos due to the copyright of the video clips used in this experiment. (**C**) Experimental protocol. Subjects performed a task of selecting one of the TV channels using the P300 BCI in four different postures. A block corresponds to one selection of a target stimulus.

The postures used in the experiment were selected from a simple preliminary online survey in which a question asked, “Which posture do you usually take when you watch TV?”. Fifty-five people participated in the preliminary survey. We selected the four most frequently submitted responses as a set of postures designed for the experiment: sitting, supine, recline, and right lateral recumbent postures (Figure 1A). Each posture was set up using the angle of the foldaway bed. The foldaway bed was 186 cm in length, 57 cm in height, and 53 cm in width, and the backside of the bed could be freely adjusted manually from 10° to 80° on a sagittal plane. Accordingly, each posture had a different angle between a vertical axis on the sagittal plane and a flat floor: 80° for the sitting posture, 45° for the reclining posture, and 10° for both the supine and right lateral recumbent postures (Figure 1A).

An eye-level in the sitting posture was set in the center of the TV screen. The eye-level in the reclining posture was 17.05 cm lower and that in the supine and right lateral recumbent postures was 49.80 cm lower than the one for the sitting posture. Meanwhile, the distance between subjects’ heads and TV was kept being 180 cm for every posture. For the sitting, recline, and supine postures, subjects leaned back against the bed with their hands stacked and placed on the legs, and their feet stretched out. For the right lateral recumbent posture, subjects turned their body to the right side from the supine posture, stacked and placed their hands on the head side. For the recline, supine, and right lateral recumbent postures, a cushion was placed below the neck to secure an angle appropriate for gazing stimuli and to prevent the electrodes from being squashed by the bed and subjects’ body.

### 2.3. Online BCI Control Task

The main task of subjects was to choose the TV channels using a P300-based BCI. The TV size was 102 cm in length and 58 cm in height. The experiment consisted of a training session followed by a test session. In both sessions, four channels displaying four different video clips were presented simultaneously at each corner of the TV screen. Every channel was displayed in a rectangular shape of the same size on black background. The video clips in each channel were randomized across blocks. The types of videos consisted of the entertainment program, documentary, news, and drama. The visual angle of the channel screen and the stimuli assigned to each channel slightly differed among the postures due to different levels of the eyes, as depicted in Figure 1B.

The stimulus presentation proceeded as follows (Figure 1C). First, a white fixation cross was shown for 0.5 s. Then, four video clips were randomly displayed at each channel for two seconds where one of the channels was set as a target and marked with a yellow border. Next, four additional red stimuli surrounding video clip windows randomly flickered one at a time, each for 10 times. Subjects were instructed to gaze at the stimulus at the target channel. The ratio of the flickering number of the target stimulus to that of the non-target stimuli was 1:3. The interstimulus interval (ISI) and stimulus duration of flickering were both 66 ms. Finally, a feedback video clip was presented for three seconds in full-screen size. The feedback video clip was the target channel to be selected for the training session and the selected channel by the BCI for the test session. This procedure of TV channel presentation and selection was termed as a block in this study. A one-second pause was placed between successive blocks with an empty black screen. 

The training consisted of 40 blocks in the sitting position, and the test session contained 15 blocks for each posture, 60 blocks in total. After proceeding with all 15 blocks of one posture, the next 15 blocks of other postures proceeded. The order of the posture in the test session was randomized across subjects. At the end of the completion of 15 blocks of one posture, a post-survey was carried out. The post-survey consisted of three questions, and each question was answered with a 10-scale score between 1 and 10; 1 indicated full disagreement, whereas 10 indicated full agreement. The questions asked about interference, comfort, and familiarity of the postures as follows: Q1: Did this posture interfere when you concentrated on the target? Q2: Did this posture make you feel comfortable when watching TV? Q3: When you watch TV in real life, are you used to this posture?

### 2.4. Data Acquisition and Preprocessing

The EEG signals of subjects from 31 active wet electrodes (FP1, FPz, FP2, F7, F3, Fz, F4, F8, FT9, FC5, FC1, FC2, FC6, FT10, T7, C3, Cz, C4, T8, CP5, CP1, CP2, CP6, P7, P3, Pz, P4, P8, O1, Oz, and O2) were acquired based on a standard head-mounted EEG cap following the 10–20 system of American Clinical Neurophysiology Society Guideline 2 and transmitted to an amplifier using a commercially available wireless system (MOVE, Brain Product GmbH Germany). This wireless system included a small, lightweight transmitter and receiver. The transmitter digitized incoming raw EEG signals and sent them to the receiver attached to the amplifier. We utilized the wireless system to transmit EEG signals reliably in different postures and to minimize potential noise from cabling due to posture changes. The mastoids of left and right ears were used as ground and reference electrodes, respectively. All electrodes were measured by keeping the impedance below 5 kΩ. The wirelessly transmitted EEG signals were amplified by a commercially available EEG amplifier (actiCHamp, Brain Product GmbH, Gilching, Germany) and sampled at 500 Hz. 

EEG preprocessing proceeded as follows: (1) high-pass filtering of raw EEG data above 0.5 Hz; (2) detection and rejection of bad EEG electrodes that exhibited a cross-correlation lower than 0.4 with more than 70% of all other electrodes [23]—three electrodes were removed on average for each subject in this study; (3) removing potential noise components referenced by common average reference (CAR); (4) low-pass filtering below 50 Hz; (5) artifact removal using the artifact subspace reconstruction (ASR) method [24,25] and (6) low-pass filtering below 12 Hz for the ERP analysis.

### 2.5. Online BCI System

We built a P300-based BCI system that distinguished a target stimulus from non-target stimuli from the user’s ERPs and sent out the information of a selected channel to the TV. Firstly, for the feature extraction of ERPs, the preprocessed EEG signal was epoched as an interval from −200 to 600 ms after stimulus onset. The ERP for a target or non-target stimulus was obtained by averaging EEG amplitudes in response to the flickering of that stimulus over repetitions and baseline-corrected by subtracting the mean amplitude of baseline defined as −200 to 0 ms within the epoch. 

Next, the procedure of extracting features used to classify the target stimulus was as follows (see Algorithm 1): (1) Amplitude values from 150 to 600 ms after the stimulus onset were selected, leading to the number of points $N_{t}=0.45\times f_{s}$, where $f_{s}$ is the sampling rate. As the sampling rate was 500 Hz, this step made $N_{t}$ = 225 for each electrode. (2) Selected samples for every electrode were concatenated. This led to the feature dimension $N_{f}$ being 225 × $N_{e}$, where $N_{e}$ is the number of electrodes. Because of the detection and rejection of bad EEG electrodes in EEG preprocessing, the feature dimension could slightly differ across subjects. ERPs from one block produced four samples of data: one target and three nontargets. Since 40 blocks were completed in the training session, a total of 160 samples were used for training a classifier with 40 for target and 120 for non-target stimulus. This produced input feature D; where $D\in {\mathbb{R}}^{N_{d}\times N_{f}}$, $N_{d}$ is the number of data samples. $N_{d}$ was 160 for the training session and 4 for single selection in the online test session (60 test samples for each posture). SVM was used for classification, because it is one of the most widely used classification algorithms for BCIs [26,27,28]. We used soft margin SVM with a linear kernel, with the objective function given by:

$$\underset{w,b,\xi }{\min\;}\frac{1}{2}{\left\|\omega \right\|}_{2}^{2}+C\overset{N}{\underset{i=1}{\sum }}\xi_{i},$$

(1) $$s.t.\;y_{i}\left(\omega^{T}x_{i}+b\right)\geq 1-\xi_{i},\;\xi_{i}\geq 0,$$

$$y_{i}\left\{\begin{array}{c} -1,\;\;\;if\;the\;class\;of\;x_{i}\;is\;negative\\ 1,\;if\;the\;class\;of\;x_{i}\;is\;positive \end{array}\right.$$

where $x_{i}$ is the i-th data sample with a class label $y_{i}$, $\xi_{i}$ is a slack variable and *C* is a penalty parameter. In this study, we fixed *C* as 1. In the online test session, after a block of the stimulus presentation was finished, classification scores of four samples were compared, and one of them was selected as a target according to the following decision criterion,

(2) $$T=\underset{c}{\mathrm{argmax}}\;\omega^{T}x_{c}+b,\;c=1,\;2,\;3,\;4$$

where *T* indicates the selected target, that is, one of the four TV channels.
**Algorithm 1** A pseudo-code for extracting features and classifying the target and non-target.**Algorithm Feature Extraction and Classification****Inputs**: ERP Dataset**Outputs**: SVM model or target stimulus *T*Step1: Amplitude values from 150 to 600 ms after the stimulus onset in the ERP dataset were picked. Step2: Concatenate Picked samples for every electrode resulting in input data D, where $D\in {\mathbb{R}}^{N_{d}\times N_{f}}.$ Step3: If training session, train linear SVM with input D and corresponding labels, and return SVM model, Else, implement SVM classification with input D and return target stimulus T given by Equation (2).

### 2.6. Data Analysis

We assessed the online BCI performance by calculating accuracy, defined as the ratio of the number of successful blocks to 15 test blocks. Furthermore, a Cohen’s kappa was used as an alternative measure to the accuracy, which was interpreted as whether the target was classified as a correct class by a chance or not [29,30]. The Cohen’s kappa (κ), for the confusion-matrix *M* including a total m-class for all subjects in each posture, was obtained as follows (see Tallón-Ballesteros and Riquelme [31]):

(3) $$\kappa =\frac{N\times trace\left(M\right)-\sum_{i}^{m}{C_{i}}_{true}\;{C_{i}}_{pred}}{N^{2}-\sum_{i}^{m}{C_{i}}_{true}\;{C_{i}}_{pred}}$$

where *N* indicates the total number of the test blocks for all subjects, and ${C_{i}}_{true}$ and ${C_{i}}_{pred}$ indicate the column and row marginal values for *M*, which implies the number of selected targets by the classifier and an expected target for class i, respectively.

Moreover, we analyzed two ERP components including P300 and N200. The ERP component P300 and N200 were obtained by the same processing for taking the ERP in an online BCI system. The EEG data from F3, FZ, F4, FC1, FC2, C3, CZ, C4, CP1, CP2, P3, PZ, and P4 was used for the analysis of P300, and those from P7, P3, PZ, P4, P8, O1, OZ, and O2 was used for the analysis of N200 [32]. Area amplitude and latency were used as the features of each component [33]. Area amplitude was derived by calculating an integral value of the positive area between 300 and 550 ms for P300, and an integral value of the negative area between 200 and 450 ms for N200. The latency was measured as the time point at 50% of area amplitude. A statistical evaluation of differences of each of these four measures (i.e., area amplitude and latency of P300 and N200) among the postures was conducted using Friedman’s test, followed by a post-hoc Dunn’s test if necessary. 

We examined a possible relationship between the subjective evaluation of a posture and BCI performance in the corresponding posture. To this end, we collected subjective responses to each post-survey question for every posture from all subjects. Since the first question of the post-survey was in opposite direction to the other two questions, higher scores in the first question indicated more negative responses, whereas higher scores in the other two indicated more positive responses; we reversed the response scores of the first question by: 10 − {original score} + 1. Then, for each question, we constructed a distribution of response scores (1~10) and calculated the median value. Note that there were 56 score values in each distribution (four postures × 14 subjects). Using the median as a threshold, we divided these responses into a high-scoring and low-scoring group, independently for every question. We then grouped 56 BCI control accuracy values corresponding to each response in the same way. Since the level of BCI performance varied across subjects, we normalized BCI control accuracy in each subject to examine the relationship of subjective attitudes to a posture with relative accuracy (e.g., to examine if a more positive response to a posture in a subject would be related to relatively higher accuracy of that subject in the corresponding posture). Normalization was performed by subtracting the average accuracy of four postures from the accuracy of each posture in each subject as follows:

(4) $$A_{ij}^{*}=A_{ij}-\frac{1}{4}\overset{4}{\underset{j=1}{\sum }}A_{ij}$$

where $A_{ij}$ and $A_{ij}^{*}$ indicate the original and normalized accuracy of subject i in a posture j, respectively. We compared accuracy between the groups using the Wilcoxon rank–sum test for each question.

## 3. Results

### 3.1. Online BCI Performance 

**Figure 2:**
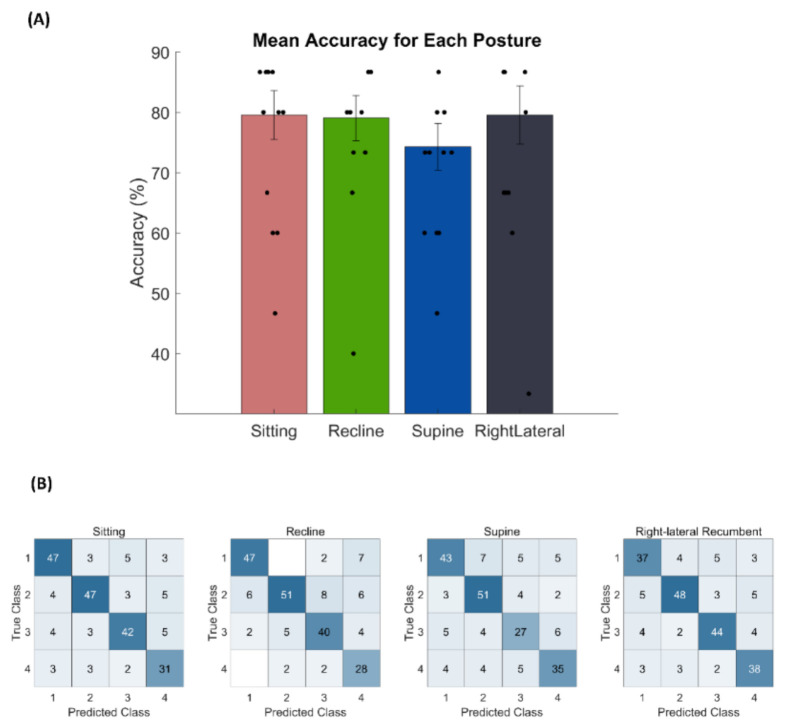
(**A**) The mean accuracy of online BCI control for each posture: sitting, recline, supine, and right lateral recumbent posture. The vertical black line denotes the standard error of the mean. The single dot represents the accuracy for each subject in each posture. (**B**) The confusion matrix for all subjects in each posture. It has 4-class for true and predicted class, which interprets as the number of selected targets for classifier and expected targets for each class, respectively. The blue color highlighted the number of targets.

The mean (±standard deviation) accuracy of online BCI performance for the sitting, recline, supine, and right lateral recumbent postures were 79.5% (±15.22), 79.0% (±14.00), 74.3% (±14.44), and 79.5% (±18.08), respectively (Figure 2A). No significant difference in accuracy among the postures was found (*p* > 0.05, Friedman’s test). Individual BCI performance with each posture is reported in Table 1. Additionally, there was no effect of the order of the test blocks on BCI performance (*p* > 0.05, Friedman’s test). Additionally, Figure 2B showed the confusion-matrix for all subjects in the test session for each posture. The obtained κ from the confusion-matrix for sitting, recline, supine, and right lateral recumbent postures were 0.726, 0.718, 0.654, and 0.726, respectively.

### 3.2. ERP Characteristics

**Figure 3:**
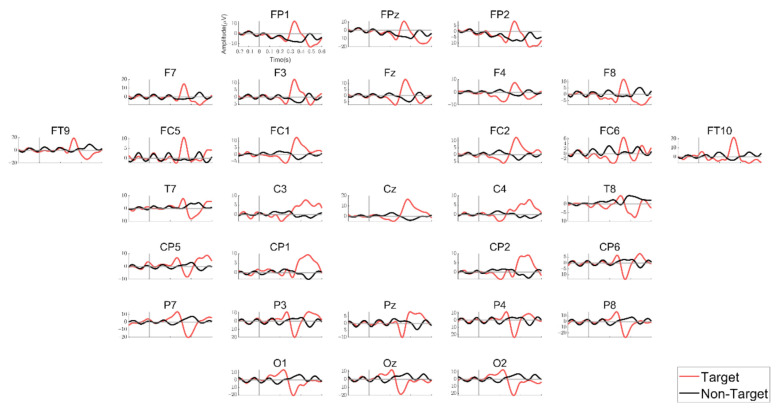
The grand averaged ERP across all subjects for the training session. The red line indicates ERP for the target stimulus and the black line denotes ERP for the non-target stimulus.

We examined the ERPs in response to the target and non-target stimuli during the training session. Figure 3 shows the grand averaged ERPs across all subjects over all the electrodes, with a clear difference in ERP patterns between the target and non-target stimuli. The target stimulus apparently elicited the P300 component in most of the electrodes, whereas the non-target stimulus did not. 

**Figure 4:**
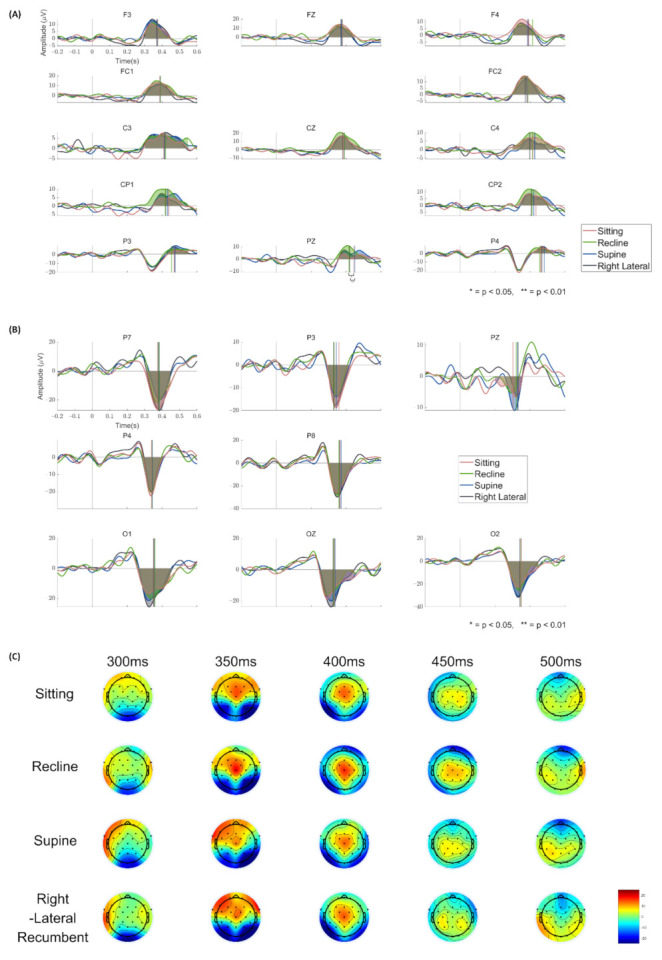
The grand averaged ERP graph for each posture. The red, green, blue, and black line indicates the ERPs for the sitting, recline, supine, and right lateral recumbent posture, respectively. The vertical lines denote the 50% area latency for each component. (**A**) The grand averaged ERP at electrodes where the P300 component appears. The shaded area represents the positive area of the ERP waveform between 300 to 550 ms after the stimulus onset. (**B**) The grand averaged ERP at electrodes where the N200 appears. The shaded area indicates the negative area of the ERP waveform between 200 to 450 ms after the stimulus onset. * *p* < 0.05, ** *p* < 0.01. (**C**) The topoplot for each posture in time 300, 350, 400, 450, and 500 ms. The color indicates the amplitude (μV).

The ERPs for the target stimulus in four different postures are illustrated in Figure 4. Figure 4A depicts prominent P300 components shown at the selected electrodes. We found no significant difference in the area amplitude of P300 among the postures at any electrodes (*p* > 0.05, Friedman’s test). Additionally, there was no difference in P300 latency among the postures at every electrode (*p* > 0.05, Friedman’s test), except in Pz (*p* < 0.0033, Friedman’s test). The post-hoc test revealed that P300 latency in the supine posture was significantly slower than the ones in the recline and right lateral recumbent postures. (supine > recline, *p* = 0.0155; supine > right lateral recumbent, *p* = 0.0074, Dunn’s test). Figure 4B shows the N200 component in the selected electrodes, and there was no significant difference among the postures in both area amplitude and latency at any electrodes (*p* > 0.05, Friedman’s test). The topoplots for each posture showed in Figure 4C. Additionally, the area amplitude and latency for P300 and N200 at each selected electrode are summarized in Table A1 and Table A2, respectively.

### 3.3. Relationship between Survey Responses and BCI Accuracy

**Figure 5:**
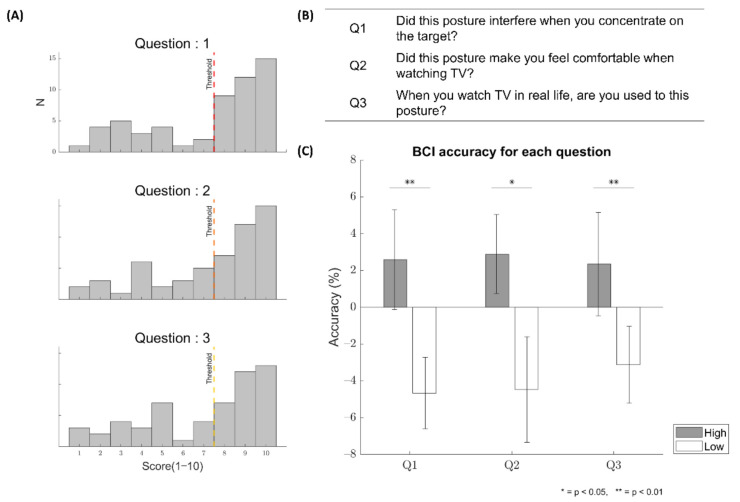
(**A**) The distribution of the scores (1–10) responded by subjects for each question. A threshold for each question was defined as the median score for the questions. N denotes the number of subjects. (**B**) Questions were asked to the subjects in the post-survey. (**C**) The average normalized BCI accuracy of the high-scoring group and low-scoring group for each question. The black vertical line denotes the standard error of the mean. * *p* < 0.05, ** *p* < 0.01.

The distributions of the scores (1~10) responding to each of the three questions in the post-survey are depicted in Figure 5A. The median score was 8 for all questions. The average response score over subjects for each posture in each question was reported in Table 2. Note that the scores for Q1 were reversed in Table 2. There was no significant difference in the response score among the postures in Q3 (*p* > 0.05, Friedman’s test), whereas significant differences were observed among the postures in Q1 and Q2 (*p* < 0.05, Friedman’s test). The post-hoc pairwise Dunn test showed differences for Q1 in the way of: sitting > supine, sitting > right lateral, and recline > supine (*p* < 0.05). The same test showed a difference for Q2 by recline > supine (*p* < 0.05). The average normalized BCI accuracy value was 2.59% (±2.71), 2.89% (±2.15), and 2.34% (±2.86) for Q1, Q2, and Q3, respectively, in the high-scoring group, and −4.67% (±1.95), −4.47% (±2.87), and −3.12% (±2.09), respectively, in the low-scoring group (see Figure 5C). There was a significant difference in normalized accuracy between the high-scoring and low-scoring groups for every question (Q1: *p* = 0.0051; Q2: *p* = 0.0142; Q3: *p* = 0.0080, Wilcoxon rank–sum test). 

## 4. Discussion

**Figure 6:**
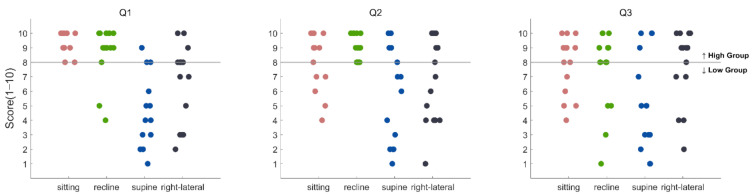
Scores for each question in each posture (sitting, recline, supine, and right lateral recumbent postures). The grey line shows a threshold for dividing the high-scoring group and the low-scoring group. Scores equal to or higher than the threshold were included in the high-scoring group.

As daily tasks are conducted in each different static posture, and the translation to the daily living environment has been one of the primary goals in the development of BCIs, this study investigated the effect of static posture on the operation of a P300-based BCI. In the online BCI experiment, subjects performed the task of controlling TV channels using the P300-based BCI system in four different postures that were likely most frequently taken when watching TV in daily life. Although the post-survey showed that the supine posture was less comfortable and more interfering in BCI operation than other postures, the BCI performance was not different among the postures (*p* > 0.05). Similarly, the area amplitude and latency of N200 and P300 in most EEG electrodes showed no difference among postures (*p* > 0.05). However, when we examined BCI control accuracy depending on subjective evaluation of postural comfort, accuracy was higher in the posture for which subjects responded more positively. This result suggests that while the physical postures employed in this study did not significantly affect the BCI performance and relevant ERP components, individual accommodations to each posture could make a difference in the BCI performance. Additionally, although the supine posture was mostly included in the low-scoring group, other postures were included in the low-scoring group to some extent depending on the questions (see Figure 6). 

There was no significant effect of the postures on the performance of P300-based BCIs as well as relevant ERP components such as P300 and N200. We examine that ERPs related to the oddball task were well evoked in each posture with no order effect and no difference between postures in the signal-to-noise ratio (SNR) using the formula derived by Rohde et al. [34], except in T7 electrode between sitting and reclining postures. These results were not in line with previous reports on the effect of posture changes on physiological states. For instance, Jones et al. mentioned that changes in postures affected the gravitational gradient on the cardiovascular and cardiopulmonary systems [10].

Several other studies reported that the hemodynamic status in the body was changed by the static posture [10,11,12,13]. Thus, our results might indicate that, despite a possible change of hemodynamic status by the change of postures, such physiological changes did not seem to influence neural responses in the oddball task significantly. Rather, our results may be consistent with an early report that no correlation was found between the change of the P300 component and the change of heart rate and blood pressure [35].

In another aspect, we found that the P300-based BCI performance differed according to subjective evaluation of postures. When subjects reported more positive feelings toward a given posture in the post-survey, their BCI performance tended to be higher than average (Figure 5C). We found this tendency in all the questions regarding interference, comfort, and familiarity. It implies that as subjects used the P300-based BCI in more comfortable and familiar postures, their BCI performance was likely to improve. 

Although it would be plausible that subjects evaluated more positively to the post-survey because they felt better after having performed BCI control more successfully in a particular posture, regardless of their genuine comfort level, it would be also plausible that using the BCI in a more comfortable posture could help subjects pay more attention to the task. In an additional analysis, we compared the area amplitude and latency of N200 and P300 between the high-scoring and low-scoring groups and found a difference at several electrodes. For example, the area amplitude of P300 at FC2 and that of N200 at P7 and P3 were larger in the high-scoring group than the low-scoring group in Q1 (*p* < 0.05, Wilcoxon’s rank-sum test). Additionally, the area amplitude of P300 CP2 was larger in the high-scoring group in Q2 (*p* < 0.05). Yet, additional studies should follow to pinpoint how comfort postures can help to operate a P300-based BCI. 

Despite using a P300-based BCI, we also observed N200, another ERP component, due to the following reason. When the visual motion stimuli were presented, N200 was elicited, especially from the temporal, occipital, and parietal regions at about 200 ms after stimulus onset (but its latency varies depending on the stimulus conditions) [36,37]. It was used to design a visual motion-based BCI, named “N200-speller”, which was similar to the P300-speller [32,38]. Since our BCI design presented the visual stimuli together with video clips that included motions, we could observe N200 as well as P300 and expect that N200 could be used as a feature used in the classification between a target and non-targets. In fact, N200 was observed in the temporal and occipital regions in our results (see Figure 3 and Figure 4B).

In a real-life BCI, the dry or semi-dry electrodes for EEG recording would be more preferable to the wet electrodes because of their quick setup and user-friendliness [39]. Although this study reported the effect of postures based on the wet electrodes, it is expected that the effect of different static postures on the P300-based BCI is likely similar when using the dry or semi-dry electrodes. However, other issues regarding the use of dry or semi-dry electrodes in different static postures might be raised. For example, Wang et al. described that some subjects experienced pain after one hour of wearing the semi-dry electrodes, which could be aggravated by extra pressure at specific postures [40]. Additionally, the contact between dry/semi-dry electrodes and skin influenced the electrode–skin impedance, influencing the signal quality [39,41,42]. Therefore, when a head position is changed by different static postures, the contact between them may bring the risk of discomfort and the change of electrode–skin impedance. Thus, it seems to be necessary to investigate the possible effects of postures on the use of BCIs with the dry/semi-dry electrodes in a wide range of aspects.

A previous study by Rice et al. reported that the thickness of the CSF layer between brain tissue and skull varies according to the posture. As the CSF layer becomes thinner, the magnitude of ERPs increased [9]. Unlike the previous study, our result did not show a difference in both the BCI performance and the ERP components among different postures. However, different from Rice et al., the head position in each posture remained the same across postures when subjects performed the task in our study. Additionally, the posture was not maintained for a long time in our study. These differences brought a limit in our study, since the duration of each posture during BCI control sessions might not be long enough to induce changes in the thickness of the CSF layer. This limitation gives rise to a need for a future study to investigate the effects of postures for the long-term use of BCIs.

Additionally, this study did not control the visual field for each posture when gazing at visual stimuli, and the number of the subjects was relatively small to verify the generalizability of experimental results. Further studies should be conducted under the control of the position of the eyeball to gaze (e.g., using an AR device) with larger sample size. In addition, the subjects who participated in this study consisted of predominantly male subjects ages 18–25 years old. However, this sample could be less representative of the relevant larger population. It also seems to be necessary to investigate the effect of different postures on the performance of P300-based BCIs in older populations. Moreover, while all subjects had the training session with equal length in the same sitting posture, certain subjects performed BCI control worse than others. This raises an important issue of how to optimize the amount of training for individuals, especially for improving the performance of poorer performers.

Nonetheless, to the best of our knowledge, this is the first study reporting the effect of postures on the online performance of P300-based BCIs. The current results at least suggest that BCI performance may not be much affected by postures per se, but rather affected by postural comfort felt by the BCI user. We envision that our results may provide useful information to design practical P300-based BCIs to be associated with smart home applications in daily living environments. Moreover, since the performance of P300-based BCIs is not significantly affected by static posture, no serious problem is expected about the use of P300-based BCI in daily life with changes in postures, thus helping to increase the possibility of commercialization of P300-based BCIs. Additionally, this study may offer a guideline that although it is possible to use a P300-based BCI in any posture, but if possible, it is recommended to maintain a comfortable posture when using the P300-based BCI system. Moreover, when the user’s BCI performance is lower than usual, the change in posture towards a more comfortable way would be one of the feedbacks to improve the performance. In clinical environments, when patients lying in bed use a P300-based BCI to control their nursing bed, their posture will change according to the control of the bed (e.g., lying to reclining). Yet, it may not affect using the P300-based BCIs such that the patients could control the nursing bed regardless of the posture.

## Figures and Tables

**Table 1:** The individual accuracy of online BCI control for each posture.

Subject	Accuracy (%)			
	Sitting	Recline	Supine	Right Lateral
‘sub1’	86.7	80.0	60.0	86.7
‘sub2’	93.3	93.3	100.0	93.3
‘sub4’	46.7	80.0	73.3	93.3
‘sub6’	80.0	40.0	60.0	60.0
‘sub7’	100.0	100.0	100.0	100.0
‘sub8’	86.7	86.7	86.7	93.3
‘sub9’	80.0	86.7	73.3	80.0
‘sub10’	66.7	80.0	80.0	66.7
‘sub11’	86.7	80.0	73.3	100.0
‘sub12’	100.0	73.3	73.3	66.7
‘sub13’	60.0	66.7	73.3	86.7
‘sub15’	60.0	73.3	60.0	33.3
‘sub16’	86.7	73.3	46.7	86.7
‘sub17’	80.0	93.3	80.0	66.7
Average	79.5	79.0	74.3	79.5

**Table 2:** The averaged response score over subjects for each question in each posture (mean ± standard deviation).

Question	Posture			
	Sitting	Recline	Supine	Right Lateral
Q1	9.43 (±0.73)	8.64 (±1.80)	4.43 (±2.44)	6.50 (±2.67)
Q2	8.07 (±1.91)	9.14 (±0.83)	5.71 (±3.17)	6.50 (±2.80)
Q3	7.79 (±1.97)	7.36 (±2.69)	5.29 (±3.24)	7.71 (±2.52)

## Data Availability

The data presented in this study are available on request from the corresponding author. The data are not publicly available due to data privacy restrictions.

## Appendix A

**Table A1:** Area amplitude (μV ∗ s) and 50% area latency (s) for the P300 in each posture (mean ± standard error).

Electrode	P300 Area Amplitude (μV ∗ s)				P300 50% Area Latency (s)			
	Sitting	Reclining	Supine	Right Lateral	Sitting	Reclining	Supine	Right Lateral
F3	1.486 (±0.260)	1.348 (±0.200)	1.571 (±0.227)	1.662 (±0.240)	0.375 (±0.0081)	0.375 (±0.0076)	0.372 (±0.0070)	0.370 (±0.0085)
FZ	2.038 (±0.227)	1.645 (±0.223)	1.237 (±0.192)	1.462 (±0.199)	0.379 (±0.0083)	0.379 (±0.0078)	0.374 (±0.0074)	0.371 (±0.0079)
F4	1.798 (±0.213)	1.169 (±0.164)	1.199 (±0.258)	1.090 (±0.171)	0.389 (±0.0119)	0.415 (±0.0181)	0.386 (±0.0139)	0.392 (±0.0173)
FC1	1.907 (±0.361)	2.073 (±0.290)	1.518 (±0.174)	1.584 (±0.195)	0.387 (±0.0088)	0.391 (±0.0068)	0.389 (±0.0072)	0.389 (±0.0085)
FC2	1.991 (±0.218)	1.899 (±0.225)	1.530 (±0.204)	1.408 (±0.125)	0.385 (±0.0068)	0.389 (±0.0078)	0.383 (±0.0078)	0.373 (±0.0050)
C3	1.394 (±0.265)	1.556 (±0.242)	1.435 (±0.215)	1.614 (±0.164)	0.411 (±0.0098)	0.420 (±0.0140)	0.416 (±0.0109)	0.411 (±0.0114)
CZ	2.067 (±0.421)	2.732 (±0.405)	1.802 (±0.206)	1.752 (±0.159)	0.384 (±0.0073)	0.391 (±0.0061)	0.391 (±0.0067)	0.381 (±0.0067)
C4	1.459 (±0.295)	1.607 (±0.234)	1.087 (±0.216)	1.041 (±0.170)	0.417 (±0.0120)	0.414 (±0.0075)	0.427 (±0.0145)	0.399 (±0.0089)
CP1	1.366 (±0.347)	1.783 (±0.342)	1.262 (±0.198)	1.080 (±0.175)	0.438 (±0.0149)	0.418 (±0.0087)	0.432 (±0.0090)	0.422 (±0.0133)
CP2	1.522 (±0.363)	1.729 (±0.267)	1.364 (±0.187)	1.080 (±0.183)	0.434 (±0.0136)	0.412 (±0.0070)	0.422 (±0.0091)	0.407 (±0.0099)
P3	0.789 (±0.179)	1.092 (±0.219)	1.108 (±0.150)	0.843 (±0.127)	0.470 (±0.0110)	0.454 (±0.0136)	0.474 (±0.0067)	0.475 (±0.0055)
PZ	1.228 (±0.346)	1.576 (±0.317)	1.493 (±0.270)	0.983 (±0.230)	0.444 (±0.0129)	0.417 (±0.0092)	0.450 (±0.0125)	0.420 (±0.0094)
P4	1.049 (±0.208)	1.097 (±0.176)	0.978 (±0.246)	0.942 (±0.185)	0.462 (±0.0085)	0.456 (±0.0114)	0.482 (±0.0103)	0.468 (±0.0084)

## Appendix B

**Table A2:** Area amplitude (μV ∗ s) and 50% area latency (s) for the N200 in each posture (mean ± standard error).

Electrode	N200 Area Amplitude (μV ∗ s)				N200 50% Area Latency (s)			
	Sitting	Reclining	Supine	Right Lateral	Sitting	Reclining	Supine	Right Lateral
P7	2.967 (±0.360)	2.094 (±0.200)	1.843 (±0.243)	2.584 (±0.363)	0.372 (±0.0058)	0.379 (±0.0060)	0.381 (±0.0063)	0.375 (±0.0047)
P3	1.922 (±0.384)	1.571 (±0.286)	1.556 (±0.267)	1.588 (±0.436)	0.359 (±0.0088)	0.331 (±0.0105)	0.345 (±0.0114)	0.329 (±0.0120)
PZ	1.312 (±0.253)	0.987 (±0.211)	1.274 (±0.280)	0.720 (±0.111)	0.303 (±0.0141)	0.322 (±0.0158)	0.330 (±0.0138)	0.331 (±0.0149)
P4	1.943 (±0.364)	1.884 (±0.369)	1.816 (±0.368)	1.491 (±0.158)	0.340 (±0.0116)	0.342 (±0.0055)	0.344 (±0.0051)	0.343 (±0.0046)
P8	2.783 (±0.490)	2.501 (±0.340)	2.813 (±0.417)	2.804 (±0.350)	0.366 (±0.0064)	0.359 (±0.0065)	0.371 (±0.0078)	0.362 (±0.0061)
O1	2.991 (±0.368)	3.166 (±0.436)	2.747 (±0.382)	3.132 (±0.452)	0.365 (±0.0113)	0.361 (±0.0079)	0.358 (±0.0068)	0.361 (±0.0108)
OZ	2.851 (±0.312)	2.556 (±0.407)	2.790 (±0.373)	2.902 (±0.339)	0.333 (±0.0090)	0.330 (±0.0079)	0.337 (±0.0090)	0.325 (±0.0081)
O2	3.149 (±0.539)	3.000 (±0.564)	3.643 (±0.695)	3.461 (±0.524)	0.360 (±0.0127)	0.354 (±0.0096)	0.355 (±0.0087)	0.348 (±0.0086)
